# Perceptions and attitudes of users and non-users of mental health services concerning mental illness and services in Japan

**DOI:** 10.3389/fpsyt.2023.1138866

**Published:** 2023-07-17

**Authors:** Takashi Uchino, Eriko Fukui, Youji Takubo, Momoko Iwai, Naoyuki Katagiri, Naohisa Tsujino, Haruhiko Imamura, Chiyo Fujii, Kuniaki Tanaka, Tetsuo Shimizu, Takahiro Nemoto

**Affiliations:** ^1^Department of Neuropsychiatry, Toho University Faculty of Medicine, Tokyo, Japan; ^2^Department of Psychiatry and Implementation Science, Toho University Faculty of Medicine, Tokyo, Japan; ^3^Tokyo Adachi Hospital, Tokyo, Japan; ^4^SODA Youth Mental Health Council, Tokyo, Japan; ^5^Department of Psychiatry, Saiseikai Yokohamashi Tobu Hospital, Kanagawa, Japan; ^6^Graduate School of Health and Nutrition Sciences, The University of Nagano, Nagano, Japan; ^7^Department of Community Mental Health and Law, National Institute of Mental Health, National Center of Neurology and Psychiatry, Tokyo, Japan; ^8^Akita Prefectural Mental Health and Welfare Center, Akita, Japan

**Keywords:** community, early intervention, integrated care system, mental health, perception, implementation research

## Abstract

**Objectives:**

There is a global movement to develop and implement community-based integrated mental health systems. The present study attempted to clarify the perceptions and attitudes of users and non-users of mental health services concerning mental illness and services in Japan.

**Methods:**

A new questionnaire was developed for this internet survey. Data from 500 outpatients with depression and 500 healthy subjects were sampled according to the demographics of the Japanese population.

**Results:**

Over 90% of healthy subjects and over 70% of patients were unaware of the common age of onset or lifetime prevalence of mental illness. Over 90% of the healthy subjects and about 70% of the patients could not describe any services where they would feel comfortable discussing mental health problems. In both groups, “adolescents and young adults” were ranked first as a target population for mental health and illness policies. The top requirement for the integrated care systems was the promotion and awareness of correct knowledge of mental illness in both the healthy subjects and patients.

**Conclusion:**

Societal requirements could include disseminating correct knowledge, awareness-raising actions for society, and implementing services where people, especially young people, can easily consult and receive support in the community.

## Introduction

Mental illnesses have an enormous impact worldwide, including increased mortality rates and social and economic losses (1, 2). In 2013, the World Health Organization (WHO) adopted the Mental Health Action Plan 2013–2020 (3). The action plan has now been extended until 2030 (4). It takes a comprehensive and multisectoral approach, through coordinated services from the health and social sectors, with an emphasis on promotion, prevention, treatment, rehabilitation, care and recovery. One of the major objectives of this action plan was to provide comprehensive, integrated, and responsive mental health and social care services in community-based settings. Efforts to implement these services have been started in various countries (5).

In Japan, the number of people admitted to psychiatric hospitals has been much higher than in other industrialized countries, and the lengths of stays have also been relatively long (6, 7). The Japanese Ministry of Health, Labour and Welfare (MHLW) decided to address mental illness intensively as one of five priority diseases, along with cancer, stroke, acute myocardial infarction, and diabetes mellitus, in the sixth revision of the Medical Care Plan from 2013. In 2019, the MHLW also announced a vision of establishing a “Community-based Integrated Care System for Mental Disorders.” The aims of this system were defined so as to ensure comprehensive medical care, welfare for disabilities, housing, social participation, employment, community cooperation, and education, enabling all individuals to live their own life as a member of a community with peace of mind, regardless of whether they have a mental illness or not and regardless of their level of disability. To promote the establishment of this system, fourteen operational items have been listed by the MHLW as of June 2022 (Table 1). Each local government can select the items and determine the contents of the services according to the actual situation in their area.

**Table 1:** Operational items listed by the Japanese Ministry of Health, Labour and Welfare to promote the establishment of a “Community-based Integrated Care System for Mental Disorders” as of June 2022.

1.	Establishment of a conference platform for health, medical and welfare professionals
2.	Project related to dissemination and awareness-raising
3.	Project to support families of persons with mental disabilities
4.	Project to support the securement of housing for persons with mental disorders
5.	Project for the inclusion of peer support
6.	Project to support outreach methods
7.	Project to support the continuation of post-discharge medical care and other services for inpatients who are at risk of self-harm or violence
8.	Project for the utilization of supporters for the establishment of a care system
9.	Project for psychiatric treatment consultation
10.	Project for the establishment of a medical coordination system
11.	Project for training staff involved in the hospital discharge and community settlement of persons with mental disorders
12.	Project to support community life for inpatients with psychiatric disorders
13.	Project for the assessment of the development of a community-based integrated care system
14.	Other projects contributing to the establishment of a community-based integrated care system

The establishment of a Community-based Integrated Care System for Mental Disorders in Japan is now in progress. The MHLW is currently reviewing operational items to ensure that they are consistent with the principles of this system. As indicated earlier in the aims of this system, this system targets the whole community, not just people with mental illness. In the present study, we aimed to clarify the perceptions and attitudes of users and non-users of mental health services regarding mental illnesses and services in Japan.

## Methods

### Study design and participants

This study had a cross-sectional design, in which data from 500 outpatients with depression and 500 healthy subjects were collected through a web-based, self-administered questionnaire survey. This survey was conducted in March 2021 by a professional agency with a large internet survey panel (Rakuten Insight, Inc., Tokyo, Japan; https://member.insight.rakuten.co.in/). A link to the online question form was distributed via email to those who had been registered on the agency’s panel. People who had been previously enrolled in the survey panel as subjects with self-reported depression or with no self-reported history of mental illness were included in the present study. The registration information for this panel was regularly checked and updated by the agency.

The participants in both groups were between the ages of 20 and 59 years and lived in Japan. The patients with depression had received continuous outpatient treatment for at least 1 year and had no history of psychiatric hospitalization within 3 months of the survey. The healthy subjects had no history of a psychiatric visit. The exclusion criteria for both groups were a history of alcohol or substance abuse and a history of brain injury, convulsive seizure, or severe physical illness. To confirm these criteria, self-reported screening questions were set prior to the main survey instruments. For the patients with depression, we asked whether the participants had been informed by a clinician that he or she had depression; if they had not, they were excluded. For the healthy subjects, we asked whether the participant had ever visited a psychiatrist for their mental health problems; if they had, they were excluded. The other exclusion criteria mentioned above were also confirmed using appropriate questions. To lessen a selection bias, the age distribution and sex ratio of each group was selected to reflect those of the latest Population Census of Japan.^1^ In addition, we excluded respondents who provided the same answer to all the questions. Furthermore, similar to the methodology used in a previous internet survey study (8), we included a question designed to detect fraudulent responses.

This study was performed as part of a research project called MEICIS (Mental health and Early Intervention in the Community-based Integrated care System), which was supported by Health Labour Sciences Research Grants (19GC1015 and 22GC1001) (9–12). Informed consent was obtained before the participants responded to the questionnaire, and the participants were given the option to stop the survey at any point. The study protocol was approved by the Ethics Committee of the Faculty of Medicine, Toho University (A20076). The internet survey agency respected the Act on the Protection of Personal Information in Japan. This study was performed in accordance with the latest version of the Declaration of Helsinki.

### Measures and data analysis

A survey questionnaire was developed by the members of the MEICIS project, which included the authors of the present report. The questionnaire was comprised of 6 fixed response or yes/no questions. The questions covered the following topics: Q1) whether the participants have knowledge of services where they can seek help comfortably; Q2) whether the participants think the current system provides early access to consultation and support services for mental health; Q3 and 4) whether the participants have knowledge of the common onset age and prevalence of mental illness; Q5) who the policy target for community-based integrated mental health services should focus on; and Q6) what specific services should be implemented in community-based integrated mental health systems. For Q6, the available choices were set to content related to early intervention in addition to choices for content specified in existing policies (Table 1). This questionnaire used simple Japanese to enable easy readability and comprehension.

The *t*-test was used to examine the differences in the ages and years of education between the groups. Regarding the results of questions about the perception of current healthcare systems and knowledge of mental illness, the chi-squared test was used to examine the differences between the two groups. Statistical differences were determined using two-tailed tests and a significance level of *p* < 0.05. A descriptive analysis was used to examine the characteristics of the answers. Data were analyzed using SPSS, version 26.0.

## Results

A total of 1,000 subjects (500 patients with depression and 500 healthy subjects) were sampled by controlling the age distribution and sex ratio of each group to reflect the Japanese population, excluding 86 subjects (7.9%) who provided fraudulent responses. The mean (standard deviation) age of the patients was 41.4 (10.5) years in the patient group and 41.4 (10.6) years in the healthy subject group. There were no significant differences in age between the two groups. Each of the two groups consisted of 254 males and 246 females (50.8 and 49.2%, respectively). The mean (standard deviation) years of education was 14.1 (2.2) years in the patient group and 14.5 (2.0) years in the healthy subject group, being significantly longer in the healthy subject group (*p* = 0.01).

**Figure 1:**
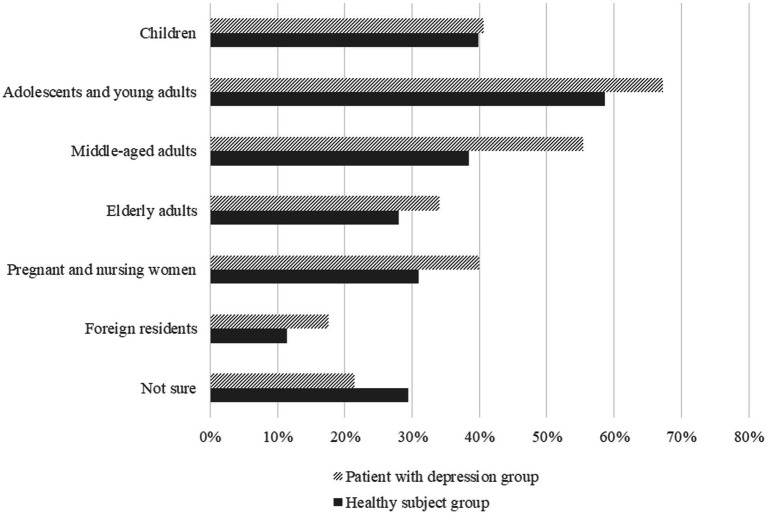
Results of the question regarding policy targets for community-based integrated mental health systems.

Table 2 shows the results of the questions about perception of current healthcare systems and knowledge of mental illness (i.e., Q1-4). For all questions, the patient group was significantly more likely to answer with a “yes” than the healthy subject group. Figure 1 shows the results of the question regarding policy targets for community-based integrated mental health systems (i.e., Q5). Table 3 shows the results of the question regarding specific methods that should be implemented in the community-based integrated mental health services (i.e., Q6).

**Table 2:** Results of questions regarding perceptions of current healthcare systems and knowledge of mental illness.

	Patient with depression group *N* = 500	Healthy subject group *N* = 500	*p* value
Q1. “When you have a mental health problem, such as anxiety, depressive mood, distress, etc., can you think of a service where you would feel comfortable discussing mental health problems?”	Yes 30.8% No 69.2%	Yes 6.2% No 93.8%	< 0.001
Q2. “Do you think the current system provides you with early access to consultation and support services when you have mental health problems or develop a mental illness?”	Yes 10.2% Not sure 47.4% No 42.4%	Yes 2.8% Not sure 66.2% No 31.0%	< 0.001
Q3. “Have you ever heard that about one in five people will experience a mental illness in their lifetime?”	Yes 28.4% No 71.6%	Yes 9.6% No 90.4%	< 0.001
Q4. “Have you ever heard that about 70% of individuals who develop a mental illness do so before the age of 25 years?”	Yes 12.4% No 87.6%	Yes 5.4% No 94.6%	< 0.001

**Table 3:** Results of the question regarding specific methods that should be implemented in the community-based integrated mental health services.

Rank^a^	Patient with depression group (*N* = 500)		Healthy subject group (*N* = 500)		Choice	proportion	Choice	proportion
1	Promotion and awareness of correct knowledge of mental illness	65.4%	Promotion and awareness of correct knowledge of mental illness	51.6%
2	Employment support for people with mental illness	59.8%	Preventive support for mental health problems before the onset of illness	36.2%
3	Preventive support for mental health problems before the onset of illness	50.6%	Support for families of people with mental illness	34.2%
4	Support for people in the early years after illness onset	50.6%	Employment support for people with mental illness	33.6%
5	Support for families of people with mental illness	48.2%	Support for people in the early years after illness onset	32.4%
6	Support for people with mental illness who are at risk of self-injury or violence	44.2%	Support for people with mental illness who are at risk of self-injury or violence	27.4%
7	Securement of housing for people with mental illness	37.8%	Securement of housing for people with mental illness	23.2%
8	Support from peer staff	28.6%	Home visitation support (outreach) for people with mental illness	21.0%
9	Support after hospital discharge for people who have been hospitalized for a long time	28.0%	Support after hospital discharge for people who have been hospitalized for a long time	18.2%
10	Home visitation support (outreach) for people with mental illness	26.4%	Support from peer staff	17.6%

a Rank is based on the % of respondents who selected the choice.

## Discussion

In the present internet survey, we investigated the perceptions and attitudes of people with depression and healthy residents in Japan towards mental illnesses and mental health services. The results of Q1 suggest that most participants could not describe any services where they would feel comfortable to discuss their mental health problems. This response may not simply represent the participants’ knowledge about the available mental health services, but may also reflect their attitudes towards approaching a mental health service when they do develop a mental health problem. A number of barriers to persons seeking help for mental health problems have been identified, a representative of which is stigma. Furthermore, it is known that the stigma attached to mental illnesses is greater in Asia than in the western countries (13, 14) and that the percentage of persons with mental illnesses that utilize mental health services is lower in Japan than in western countries (15, 16). Therefore, many participants may have answered that there are no services where they would feel comfortable to discuss their mental health problems because of stigma rather than because of an actual perception of the inadequacy of the services.

Nevertheless, since the proportion of the healthy group who answered that they could think of a service was very limited, this may be due to the minimal existing mental health services in Japan and the lack of knowledge about mental health and illness. Current Japanese policy does not include the implementation of consultation services, including those for young people, the most common age for mental illness (17, 18). Policies are needed to enable local governments to implement services where people can easily consult and receive support in the community. Consultations or medical services specializing in early intervention in mental health or illness have been provided only in a few, mainly university hospitals (19–22). This challenge is not unique to Japan. Even in many high-income countries, early intervention services are far from being sufficiently widespread (23). A lack of community services can lead to serious delays in treatment (24, 25). Many individuals not only with psychosis but also with depression and neurosis remain untreated; thus, early intervention remains a serious global challenge (26–28).

Regarding basic knowledge of mental illness, only about 10% of the patient group and 5% of the healthy group were aware of the common onset age and prevalence of mental illness. Regarding the required health care system, “adolescents and young adults” was ranked as the priority target of policies on mental health and illness, and the top requirements for community-based integrated mental health care systems were the promotion and awareness of correct knowledge of mental illness, preventive support for mental health problems before the onset of mental illness, and support for people in the early years after illness onset. These requirements were selected more frequently than those regarding existing policies. These findings suggest a high level of need and social interest in primary and secondary prevention for young people, together with the dissemination of correct knowledge and awareness-raising actions for society (29). These contents, which are likely to be desired by many people, should be included in the community-based integrated care systems that are currently being constructed in Japan. The MEICIS project in Japan, funded by the MHLW from 2019, is examining and making policy recommendations on specific early intervention services to address these unmet medical needs. For example, an integrated youth mental health service that has been implemented in other countries (e.g., “headspace” in Australia) was introduced in a metropolitan area of Japan from 2019 (9). The MEICIS project has also been working on developing a system for public health nurses to enhance their skills and provide mental health care for people in need in Akita Prefecture, which is a depopulated area of Japan, using Information and Communication Technology (ICT) (Akita Mental health ICT Network, AMIN), in addition to examining the effective placement of clinical psychologists in outreach teams and considering the development of an easy-to-access system for foreign residents, who are increasing in number in Japan (10–12, 30). Furthermore, the project has adopted the methods of dissemination and implementation science to identify promoting and hindering factors of these implementations and to facilitate evidence-based practices (31, 32). The Japanese government needs to consider policies to expand the implementation of necessary services based on epidemiological evidence, as well as further raise public awareness about mental health and illness and promote the use of appropriate services. Prior cases in other countries have reported the importance of building partnerships with local communities (33, 34), and this point needs to be taken into account in the development of policies of implementation strategies that have been adapted to Japan’s unique context.

The limitations of the present study include the fact that all diagnoses were self-reported, and we were unable to investigate the severity of illness, patients with illnesses other than depression, or hospitalized patients. In addition, both groups did not include minors or elderly people. The participants in this study had relatively long years of education, given that the mean duration of education in the Japanese epidemiological study was 12.9 years. Furthermore, the healthy subjects had longer years of education than the patient group. The geographical location of the participants, which we were unable to examine in the present study, could have influenced the level of education of the subjects, as also their level of knowledge about mental illnesses. Although these limitations exist, the results were collected from a wide and large number of subjects through an internet survey, and the results were controlled for age and sex according to national demographics. In general, it is estimated that one in five persons would develop a mental illness in his/her lifetime (27, 35). However, Japan is considered to have lower income inequality than other countries, which could have influenced our estimated prevalence of mental illnesses in Japan (36). Therefore, the results of this study can only be extrapolated with caution to the situation in other countries.

In the present study, we included healthy subjects and patients with depression, however, further investigation is warranted on the perceptions about mental health services among patients with severe mental disorders (SMD), such as psychotic disorders. The social and economic losses associated with SMD are enormous, and patients with SMD are the main users of mental health services. Early interventions for psychotic disorders lead to both improved symptomatic and functional outcomes, and favorable evidence has been repeatedly reported for the effectiveness of early intervention services for these disorders (37). In addition, there is also growing evidence for a favorable cost-effectiveness of these services (38). The acceptability of these services to the patients is an important component of healthcare system development.

## Conclusion

Societal requirements could include disseminating correct knowledge, awareness-raising actions for society, and implementing services where people, especially young people, can easily consult and receive support in the community. The Community-based Integrated Care System for Mental Disorders in Japan needs to take these services into account.

## Data availability statement

The raw data supporting the conclusions of this article will be made available by the authors, without undue reservation.

## Ethics statement

The studies involving human participants were reviewed and approved by the study protocol was approved by the Ethics Committee of the Faculty of Medicine, Toho University (A20076). The patients/ participants provided their written informed consent to participate in this study.

## Author contributions

TU and TN designed the study and wrote the protocol. EF, YT, MI, NK, NT, HI, CF, KT, and TS were involved in the conceptualization of the study. TU collected the data, undertook the statistical analysis, and wrote the first draft. TN revised the manuscript. All authors contributed to the article and approved the submitted version.

## Funding

This research was supported by Health Labour Sciences Research Grants (19GC1015 and 22GC1001) to TN.

## Conflict of interest

TU and TN belong to Department of Psychiatry and Implementation Science, Toho University Faculty of Medicine, that is funded by Nippon Life Insurance Company.

The remaining authors declare that the research was conducted in the absence of any commercial or financial relationships that could be constructed as a potential conflict of interest.

## Publisher’s note

All claims expressed in this article are solely those of the authors and do not necessarily represent those of their affiliated organizations, or those of the publisher, the editors and the reviewers. Any product that may be evaluated in this article, or claim that may be made by its manufacturer, is not guaranteed or endorsed by the publisher.

## Footnotes

^1^ https://www.stat.go.jp/english/data/kokusei/2020/summary.html
